# PREDICTING OLDER ADULTS’ CONTINUED COMPUTER USE AFTER INITIAL ADOPTION

**DOI:** 10.1093/geroni/igad104.0822

**Published:** 2023-12-21

**Authors:** Shenghao Zhang, Walter Boot

**Affiliations:** Florida State University, Tallahassee, Florida, United States; Florida State University, Tallahassee, Florida, United States

## Abstract

Digital technologies have the potential to help older adults in various aspects of their lives, but older adults need to adopt and keep using the new technologies to reap the benefits. Previous research on digital divide mainly focuses on adoption and acceptance of new technologies, and little is known about what might influence actual use and disuse after this initial stage. The current study modeled changes in constructs related to computer use after initial computer adoption and examined whether these changes predict continued use. We used data from the computer arm (N = 150, MAge = 76.15) of a 12-month field trial examining the potential benefits of computer use in older adults. Individual differences identified in the technology acceptance literature (perceived usefulness, ease of use, computer interest, computer self-efficacy, computer anxiety, quality of life, social isolation, and social support) were measured before (baseline), during (month 6), and after the intervention (posttest). Univariate and bivariate latent change score models examined changes in each predictor and their potential causal relationship with use. Results demonstrated large inter-individual differences in the change patterns of individual difference factors examined. Change in perceived usefulness, perceived ease of use, and computer interest, computer self-efficacy, and computer anxiety were correlated with but not predictive of change in use. Our findings demonstrate the limitation of popular constructs in technology acceptance literature in predicting continued use after initial adoption and point out important gaps in knowledge to be targeted in future investigations.

